# Draft genome sequence of *Serratia fonticola* TU-B-200-1 isolated from the upper Taehwa River with potential for carbon fixation

**DOI:** 10.1128/mra.00864-26

**Published:** 2026-10-21

**Authors:** Gwang Il Jang, Byung Cheol Cho, Ha Yeon Kang, Gyeong Geun Park, Ha Eun Lee

**Affiliations:** 1 Department of Chemical Engineering, Ulsan College 65683, Ulsan, Korea; 2 Research Institute of Oceanography, Seoul National University 26725, Seoul, Korea; Indiana University Bloomington, Bloomington, Indiana, USA

**Keywords:** *Serratia fonticola*, genome sequence, carbon fixation, Taehwa River

## Abstract

The complete genome of *Serratia fonticola* TU-B-200-1, isolated from the upper Taehwa River, Ulsan, Korea, spans 5,832,012 bp with a GC content of 53.6% and encodes 5,201 CDs. Functional annotation revealed genes associated with carbon fixation and metabolism, suggesting a potential ecological role in carbon cycling.

## ANNOUNCEMENT

The genus *Serratia* was established by Bizio in 1823 with *Serratia marcescens* as the type species and is presently assigned to the family *Yersiniaceae* (1, 2). Members are gram-negative, facultatively anaerobic rods with broad metabolic versatility, encompassing over 24 species from diverse environments, including *Serratia fonticola*, originally described from freshwater habitats and noted for fermenting diverse sugars and tolerating varied pH and salinity conditions (3, 4). As part of ongoing surveillance of freshwater microbial diversity in the Taehwa River, an area subject to increasing anthropogenic and agricultural input, we sought to characterize culturable heterotrophic bacteria with potential biogeochemical roles. Strain TU-B-200-1 was selected for whole-genome sequencing based on its rapid growth, phylogenetic placement within *Serratia*, and potential ecological significance in this habitat. Strain TU-B-200-1 was recovered from a water sample collected from the upper Taehwa River in Ulsan (35.54°N, 129.00°E), Korea, spread onto LB agar (MB Cell, Korea), and incubated at 30°C for 48 h; pure cultures were obtained through three streak-plate rounds. DNA for 16S rRNA gene amplification was extracted by the boiling method (5) and PCR-amplified with primers 27F and 1492R. Products were sequenced (Applied Biosystems, Macrogen, Inc., Korea) and compared against GenBank and EzBioCloud using blastn (5, 6), confirming assignment to *Serratia*. Genomic DNA for sequencing was extracted from a 36-h LB broth culture (Maxwell RSC Tissue DNA Kit; Promega, USA). Libraries were prepared with the TruSeq Nano DNA Library Prep Kit (Illumina, USA) and sequenced (2 × 150 bp) on a NovaSeq X platform by Macrogen, Inc. Read quality was assessed with FastQC v0.11.7 (7). Low-quality reads (>10% bases < Q20) were discarded, and adapters were trimmed with Trimmomatic v0.38 (8). Genome size was estimated with Jellyfish v1.1.12 (9) and GenomeScope v1.0 (10). *De novo* assembly used SPAdes v3.15.0 with multiple k-mer values (11), excluding contigs shorter than 1,000 bp. Completeness was evaluated with BUSCO v5.1.3 (12). Gene prediction used Prokka v1.14.6 (13), with additional annotation via InterProScan v5.34-73.0 (14) and eggNOG-mapper v2.1.10 (15) against eggNOG DB v5.0.2 (16); counts are listed in Table 1. Species identity was validated via ANI against strain LMG 7882(T) (AVAH00000000) using pyani v0.2.12 (17). A phylogenetic tree was constructed with MEGA-X v12.0.11 (18).

**TABLE 1:** Information on the draft genome of *Serratia fonticola* TU-B-200-1

Parameters	Results
Genome size (bp)	5,832,012
GC content (%)	53.6
Total number of contigs	36
Largest contig	873,300
CDS	5,201
Number of total genes	5,289
Total number of reads	5,793,388
N50	428,087
tRNAs	78
rRNAs	9
tmRNA	1

**Fig 1:**
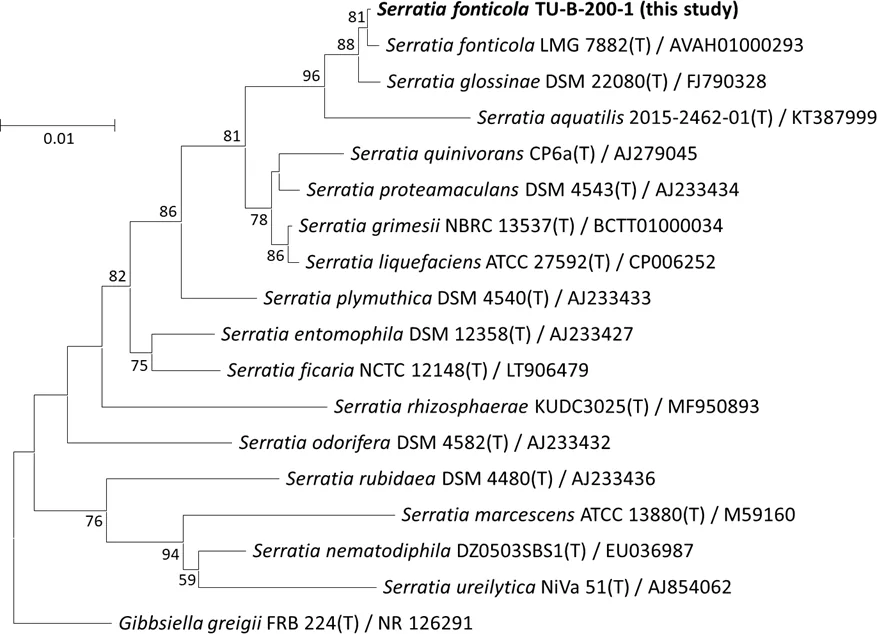
Neighbor-joining tree based on 16S rRNA gene sequences using the Kimura 2-parameter method in MEGA-X 12.0.11, showing the phylogenetic position of strain TU-B-200-1 within related genera. Bootstrap values were based on 1,000 replicates (values ≥50% are shown). *Gibbsiella greigii* FRB 224(T) was used as the out-group. Bar, 0.01 changes per nucleotide position.

Paired-end sequencing generated 22,844,984 reads, yielding 5,793,388 clean reads (Q20: 99.5%, Q30: 96.9%) at 150.60-fold coverage. The assembled genome spans 5,832,012 bp with a GC content of 53.6%. Annotation identified 5,201 CDSs, 78 tRNA genes, 9 rRNA genes, and 1 tmRNA (Table 1). The 16S rRNA gene (1,505 bp) confirms placement within *Serratia* (Fig. 1), with the closest relative being *S. fonticola* LMG 7882(T) at 99.9% sequence similarity. This was supported at the genome scale: ANI between strains TU-B-200-1 and LMG 7882(T) was 95.82% (coverage 84.9%), exceeding the 95%–96% species threshold (19). Although *Serratia* is typically chemoheterotrophic, the strain TU-B-200-1 harbors genes associated with the Calvin–Benson–Bassham cycle, including a plasmid-borne phosphoribulokinase, a carboxysome shell protein (CcmK), and multiple carbonic anhydrases, suggesting an unrecognized metabolic niche for carbon assimilation in nutrient-limited freshwater environments.

## Data Availability

The whole-genome sequencing project for *Serratia fonticola* TU-B-200-1 has been deposited at DDBJ/ENA/GenBank under accession number JCAGXA000000000 under BioProject no. PRJNA1483895. The version described in this paper is version JCAGXA010000000. BioSample accession number SAMN61208929 and SRA accession number SRR39388441 are available at NCBI.
